# Epitope definition by proteomic similarity analysis: identification of the linear determinant of the anti-Dsg3 MAb 5H10

**DOI:** 10.1186/1479-5876-2-43

**Published:** 2004-12-11

**Authors:** Alberta Lucchese, Abraham Mittelman, Mong-Shang Lin, Darja Kanduc, Animesh A Sinha

**Affiliations:** 1 Department of Odontostomatology and Surgery, Faculty of Medicine, University of Bari, P.za G. Cesare 11, 70124 Bari, Italy; 2 Department of Medicine, New York Medical College, Valhalla, NY 10595, USA; 3 Department of Dermatology, Medical College of Wisconsin, Milwaukee 53226, USA; 4 Department of Biochemistry and Molecular Biology, University of Bari, Via Orabona 4, 70126 Bari, Italy; 5 Department of Dermatology, Weill Medical College of Cornell University, 1300 York Avenue, New York, NY 10021, USA

**Keywords:** Epitope mapping, Computational biology, Proteomics, Desmoglein 3, *Pemphigus vulgaris*

## Abstract

**Background:**

Walking along disease-associated protein sequences in the search for specific segments able to induce cellular immune response may direct clinical research towards effective peptide-based vaccines. To this aim, we are studying the targets of the immune response in autoimmune diseases by applying the principle of non-self-discrimination as a driving concept in the identification of the autoimmunogenic peptide sequences.

**Methods:**

Computer-assisted proteomic analysis of the autoantigen protein sequence and dot-blot/NMR immunoassays are applied to the prediction and subsequent validation of the epitopic sequences.

**Results:**

Using the experimental model *Pemphigus vulgaris*/desmoglein 3, we have identified the antigenic linear determinant recognized by MAb 5H10, a monoclonal antibody raised against the extracellular domain of human desmoglein-3. The computer-assisted search for the Dsg3 epitope was conducted by analyzing the similarity level to the mouse proteome of the human desmoglein protein sequence. Dot-blot immunoassay analyses mapped the epitope within the sequence Dsg3_49–60 _REWVKFAKPCRE, which shows low similarity to the mouse proteome. NMR spectroscopy analyses confirmed the specificity of MAb 5H10 for the predicted epitope.

**Conclusions:**

This report promotes the concept that low level of sequence similarity to the host's proteome may modulate peptide epitopicity.

## Introduction

In the last decades, several computer-driven algorithms have been devised to take advantage of the linear representation of protein sequence information to search for epitopic motifs [1-5]. These algorithms search the amino acid sequence of a given protein for characteristics believed to be common to antigenic peptides, locating regions that are likely to induce cellular immune response. Given the rapid expansion of proteomic sequence data, the application of these algorithms to disease-associated proteins may direct research to specific segments of disease-associated proteins and thus potentially reduce the time and effort needed to develop effective vaccines [6,7].

We are using *in silico *technology platforms to identify epitopic peptide sequence(s) from disease-associated-antigens by following the hypothesis that peptide epitopicity might be regulated by the peptide similarity level to the host's proteome, in addition to other factors such as MHC binding potential [8-10]. As a scientific rationale, we follow the criterion that immune system might be allowed/forced to respond only to rarely encountered/never seen antigenic sequences. Accordingly, we explain the non-immunogenicity of tumor-associated-antigens as due to high level of similarity of oncoprotein sequences to self-proteome [8,9,12].

In this context we have tested here the possibility that endogenous, normal, housekeeping self-proteins harbouring sequences with little or no similarity to the collective host proteome might be epitopic targets in autoimmune responses. Self-reactivity and autoimmunity are processes related to the breakage of self-tolerance that can be distinguished by their different clinical outcome. The transition from self-reactivity to the autoimmune pathology appears to be mediated by a complex network of overlapping phenomena that comprehend epitope spreading, uncovering of cryptic epitopes, natural autoantibodies production, cross-reactivity, microchimerism, altered B lymphocyte function, inflammation, etc. [13]. In our labs, we are studying the autoantibody profile in *Pemphigus vulgaris *(PV). PV is an autoimmune bullous skin disease characterized by autoantibodies to a desmosomal adhesion molecule, the cadherin desmoglein-3 (Dsg3) [14]. Dsg3 represents an optimal autoantigen for studying the relationship between similarity level and immune responses. Indeed the PVA Dsg3 is a highly conserved protein, and the human and mouse forms present 71.6% of identity. Therefore, this protein allows to analyze the sequence specificity of the reaction PVA-autoantibody by using murine monoclonal antibodies. Moreover, our interest to study the autoantibody response in this autoimmune disease also stems from the following considerations: i) so far, notwithstanding the Dsg3 linear structure, attention has been focused mainly on conformational Dsg3 epitopes [15-18] and there is a lack of data on the occurrence and fine molecular characterization of linear Dsg3 epitopes; ii) although the precise pathological implications of anti-Dsg autoantibodies are not fully elucidated [19-23], it seems likely that a spectrum of autoantibodies, differing in number and type, might contribute to the autoimmune pathology in PV [24]. Given these premises, the development of new experimental approaches that might lead to the exact definition of linear PVA epitopes is desirable. As a first attempt to a computer-driven individuation of defined linear epitopic sequences of Dsg3, we describe here the identification of the linear epitope of the MAb 5H10 raised in BALB/c mice against the extracellular domain (EC1/EC2, aa1-212) of human Dsg3.

## Materials and methods

### Computer-assisted analyses

The amino acid sequence corresponding to accession number P32926, SWISS-PROT, was used in the similarity analysis of the extracellular domain of human Dsg3 (EC1 to part of EC2, amino acid 1–212) to the mouse proteome. The Dsg3 sequence under analysis was dissected into pentamer motifs, that were probed for sequence similarity to mouse proteome by using PIR non-redundant reference protein database and peptide match program [25]. The search was conducted against mouse complete genome for a total of 78435 sequences, and includes hypothetical/unidentified proteins sequences.

### Antibodies

Anti-Dsg3 MAb 5H10 and 5G11, raised in BALB/c mice, were a generous gift of Dr. Margaret Wheelock, University of Toledo, Ohio. Both MAbs recognize a linear determinant and have been described in detail [26]. Briefly, MAb 5H10 has been shown to recognize the amino terminal part of the extracellular domain of Dsg3 (EC1 to part of EC2, amino acid 1–212). MAb 5G11 reacts with the carboxyl terminus of the EC domain of Dsg3 (part of EC4 to EC5, amino acid 446–613). Horseradish peroxidase (HRP)-conjugated goat anti-mouse IgG-specific Abs was from Sigma, Chemical Co., St.Louis, Mo.

### Synthetic peptides

Peptides were synthesized using standard Fmoc (N-(9-fluorenyl)methoxycarbonyl) solid phase peptide synthesis. Peptide purity (>90%) was controlled by analytical HPLC, and the molecular mass of purified peptides confirmed by fast atomic bombardment mass spectrometry. The EC1/EC2 Dsg3_36–45_EEMTMQQAKR, Dsg3_49–60_REWVKFAKPCRE, and Dsg3_173–187 _NSLVMILNATDADEP peptides were obtained from PeptidoGenic Research & Co., Livermore, CA., and used in dot-blot immunoassay experiments. Human recombinant Dsg3 protein was used as further control [27]. The ^15^N-labelled synthetic peptide Dsg3_49–60_REWVKFAKPCRE (where ^15^N-labelled amino acid residues are underlined) was from Primm srl, Milan, Italy. Peptides were dissolved in 0.9% NaCl, aliquoted and stored at -20°C.

### Dot immunoassay

Nitrocellulose membrane (0.2 μm pore size, BioRad Laboratories, Hercules, CA) was pretreated for 10 min with 1.0% glutaraldehyde. Synthetic peptides were spotted on the activated membranes, left to dry at RT and exposed to UV rays for 10 min. Membranes were incubated for 1 h in phosphate-buffered saline/0.05% (v/v) Tween 20 (PBST) containing 4% bovine serum albumin (BSA), and then with the primary MAb (1:800) for further 2 h. Membranes were washed for 10 min with PBST (x3) and incubated in PBST/4% BSA with HRP-conjugated affinity-purified goat anti-mouse IgG (1:1000) for 1 h. Membranes were washed in PBST for 5 min (x3), in PBS for 5 min (x3) and immunoblots were developed using the enhanced chemiluminescence detection assay (Renaissance, NEN™ Life Science Products, Boston, MA.) following supplier's instructions.

### NMR spectroscopy

NMR spectra of the reaction between the synthetic ^15^N-labelled peptide Dsg3_49–60_REWVKFAKPCRE (where ^15^N-labelled amino acid residues are underlined) and MAb 5H10 (raised against EC1/EC2 from Dsg3) or 5G11 (raised against EC4/EC5 from Dsg3) were recorded at 298°K on a Bruker Avance DRX500WB spectrometer. The spectra were acquired by heteronuclear single quantum correlation (HSQC) experiments that correlate the chemical shift of proton with the chemical shift of the directly bonded nitrogen. Specifically, the two-dimensional ^1^H-^15^N inverse detected correlation spectra were acquired by the gradient pulse sensitivity improved Bruker automation program INVIETGPSI [28]. The ^1^H acquisition dimension was collected with a spectral width of 5 ppm, centered at 7.6 ppm, using 4096 datapoints for each of the total 32 scans/expt. Spectral width in the indirect detected ^15^N dimension was 200 ppm, centered at 90 ppm, obtained with a total of 1024 points. The spectra were processed by XwinNMR program, version 2.6, running on an INDY R5000 Silicon Graphics Workstation. Chemical shift values were referenced to sodium tetrasilyl propionate (TSP) (^1^H; 0.000 ppm) and external neat nitromethane (^15^N; 380.23 ppm) standards. To avoid interferences of TSP standard with peptide samples, the instrument scale was preliminarily calibrated in parallel experiments with samples containing the peptide to be analysed plus 0.2 mg TSP. We used chemical shift statistics from the full BioMagResBank database, where the calculated statistics are derived from a total of 559392 chemical shifts (website: ). Sequence-specific correction factor tabulations were applied to backbone ^1^H and ^15^N resonances [29]. Two-dimensional correlated spectroscopy spectra of MAb-peptide complex were obtained using a 1:2 stoicheiometric molar ratio (peptide:MAb, 0.1:20, mg/mg). That is, NMR samples contained either 0.1 mg free REWVKFAKPCRE peptide, or 20 mg MAb 5H10 complexed with 0.1 mg REWVKFAKPCRE peptide, or 20 mg MAb 5G11 complexed with 0.1 mg REWVKFAKPCRE peptide, in 0.5 ml aqueous solution H_2_O/D_2_O (9:1, v/v).

## Results

### Selection of Dsg3 peptide sequences with low similarity to the mouse proteome

**Figure 1:**
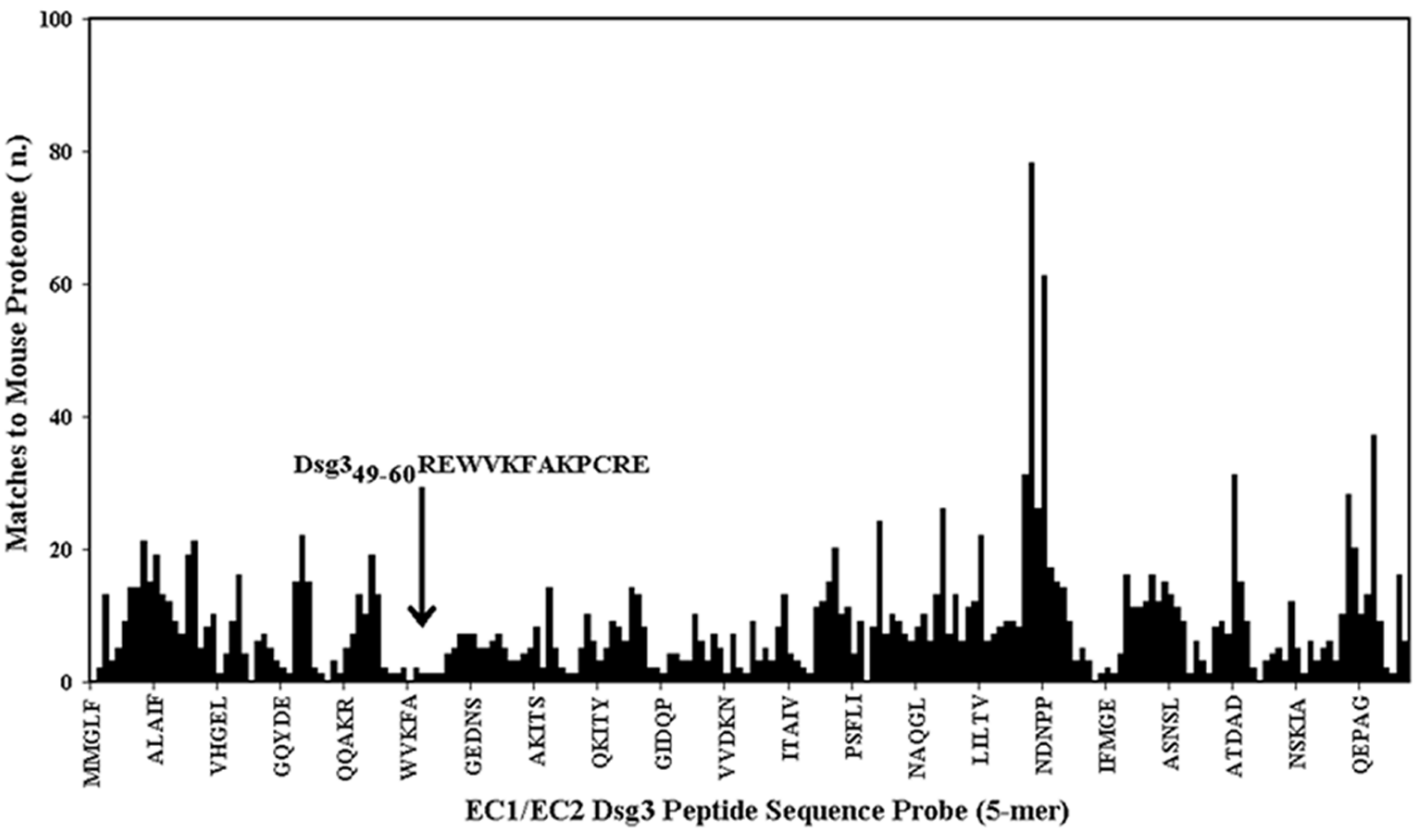
**Molecular mimicry between the EC1/EC2 of human Dsg3 and mouse proteome**. The EC1/EC2 Dsg3 sequence (aa1-212) was scanned for matches to mouse protein sequences by using pentamers offset by one residue. The arrow indicates the longest stretch having the lowest number of matches.

The EC1/EC2 domain (aa1-212) of human Dsg3 was dissected into 5-mer sequences and analyzed for similarity to mouse proteins. The pentamers used for epitope scanning were offset by one residue, i.e. overlapped by 4 residues: MMGLF, MGLFP, GLFPR, LFPRT, etc. The 5-mer sequences were probed in computer-assisted similarity analyses against the complete mouse genome sequences. Fig. 1 reports the profile we obtained by representing the number of matches to mouse proteome over the sequential pentamer peptides. It can be seen that the EC1/EC2 Dsg3 protein sequence presents stretches scarcely represented in the mouse proteome. Among the low-similarity EC1/EC2 Dsg3 peptide fragments, the Dsg3_49–60_REWVKFAKPCRE sequence was the longest (12 amino acid residues long) with the lowest number of matches to the murine proteome (number of matches to murine proteome = 9). The Dsg3_49–60_REWVKFAKPCRE sequence and a second low-similarity fragment, Dsg3_36–45_EEMTMQQAKR (number of matches to murine proteome = 12), were selected to test our hypothesis.

### Dot-blot immunoassay

**Figure 2:**
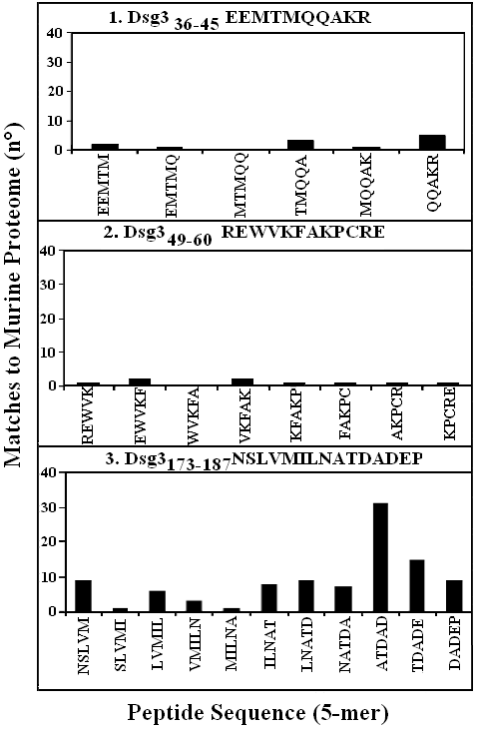
**Similarity scanning on the human Dsg3 peptide sequences selected for immunoassay analysis with murine anti-EC1/EC2 Mab 5H10**. Matching analysis to the murine proteome was performed using as probes pentamers offset by one residue as described under Methods. Peptide: 1) Dsg3_36–45_EEMTMQQAKR; 2) Dsg3_49–60_REWVKFAKPCRE; 3) Dsg3_173–187_NSLVMILNATDADEP.

**Figure 3:**
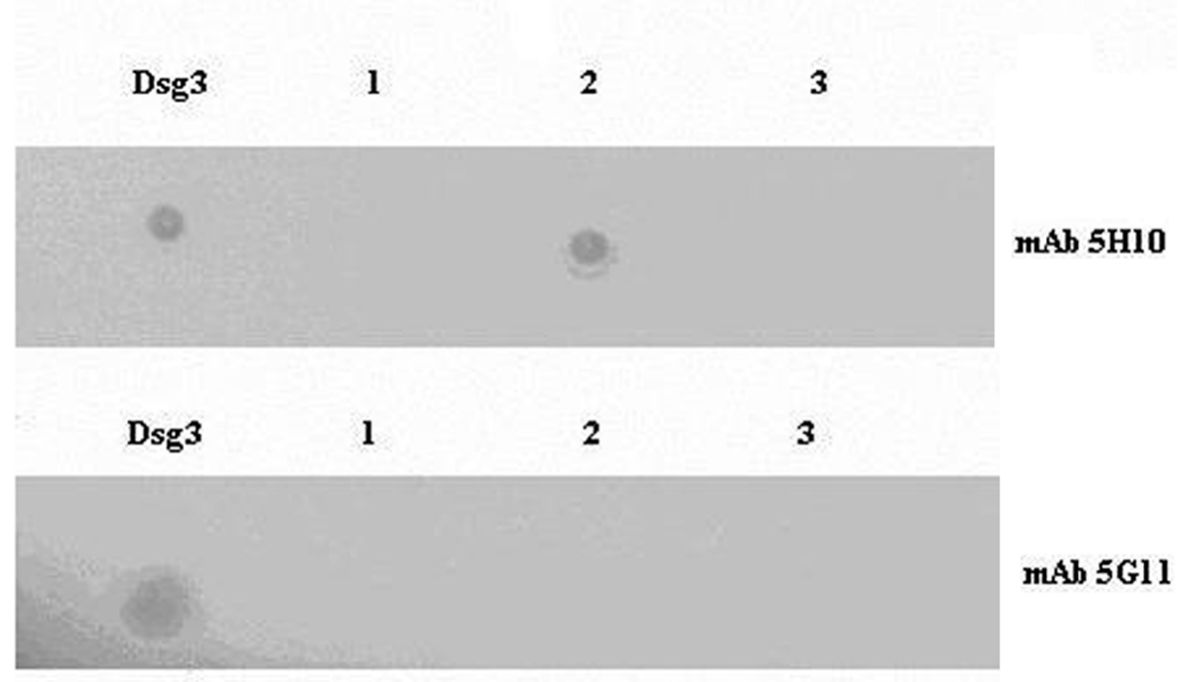
**Identification of the epitopic sequence recognized by mouse anti-Dsg3_1–212 _MAb 5H10**. Dot-blot immunoassay was performed on nitrocellulose membrane spotted with human Dsg3 (10μg), or Dsg3 peptide (2.5μg) corresponding to the sequence: 1) Dsg3_36–45_EEMTMQQAKR; 2) Dsg3_49–60_REWVKFAKPCRE; 3) Dsg3_173–187_NSLVMILNATDADEP.

The two low similarity peptides selected as described above were synthetised in order to be assayed in immunodot-blot analyses to identify the linear determinant of the MAb 5H10. In addition, the synthetic Dsg3_173–187_NSLVMILNATDADEP peptide (matches to murine proteome = 109) was available and served as a high similarity peptide control. The three synthetic peptides are described in Fig. 2. The MAb 5G11 with specificity to the terminal EC portion of Dsg3, amino acid 446–613 [26] was used as a primary antibody control. The immunodot-blot experimental result is illustrated in Fig. 3. It can be seen that MAb 5H10 recognized peptide n. 2, i.e. the Dsg3_49–60_REWVKFAKPCRE sequence having a lowest level of similarity to the mouse proteome (see Fig. 1). No reaction was monitored using MAb 5G11.

### NMR spectroscopic immunoanalysis

As a further step, we carried out a parallel experimental confirmation because of the doubts of false negative/positive data intrinsic to immunoassay. To this aim, the binding of the predicted epitopic Dsg3_49–60_REWVKFAKPCRE peptide and the mouse anti-Dsg3 5H10 MAb was further verified by NMR spectroscopy. NMR spectroscopy can be used in 1) defining structure and conformation; 2) defining structure-activity relationships; 3) monitoring chemical reactions. Informations are mainly derived by measuring NMR chemical shifts. The chemical shift of a nucleus is the difference between the resonance frequency of the nucleus and a standard. This quantity is reported in parts per million (ppm). The NMR standards are molecules as tetrasilyl propionate, the signal of which is set at 0 ppm by having shielded protons. The NMR chemical shift allows for distinguishing magnetically inequivalent nuclei in a molecule, i.e. chemical shifts are a measure of the motional freedom.

We utilized NMR spectroscopy in order to determine whether the predicted peptide specifically binds to the MAb 5H10. To this aim, a ^15^N-labelled REWVKFAKPCRE peptide(where ^15^N-labelled amino acid residues are underlined) was synthesized. Theoretical chemical shift values of the ^15^N-labelled residues in the Dsg3_49–60 _REWVKFAKPCRE peptide were calculated as described under Methods, and then were compared to the experimental values. Table 1 lists the theoretical and experimental chemical shift values of ^15^N-labelled residues in the Dsg3_49–60 _REWVKFAKPCRE peptide, and their values following addition of MAb 5H10 or MAb 5G11.

**Table 1:** Sequence-specific assignments in the ^15^N-labelled Dsg3_49–60_REWVKFAKPCRE peptide, and chemical shift changes on MAb 5H10 or 5G11 binding.

^15^N-Amino acid	Chemical Shift Values:							
	Theoretical: Free peptide		Experimental: Free peptide		+MAb 5H10		+control MAb 5G11	
		
	^1^H^N^	^15^N	^1^H^N^	^15^N	^1^H^N^	^15^N	^1^H^N^	^15^N
Arg-1	6.78	77.6	6.95	81.0	n.d.	n.d.	7.05	80.5
Val-4	8.65	120.8	8.19	122.8	n.d.	n.d.	8.18	122.5
Phe-6	8.42	121.7	7.99	119.2	n.d.	n.d.	8.00	118.9
Ala-7	8.29	122.3	8.40	120.0	n.d.	n.d.	8.44	120.2
Arg-11	6.78	77.6	7.25	81.5	n.d.	n.d.	7.26	81.0
	8.14	119.6	8.16	118.1	n.d.	n.d.	8.15	117.8

**Figure 4:**
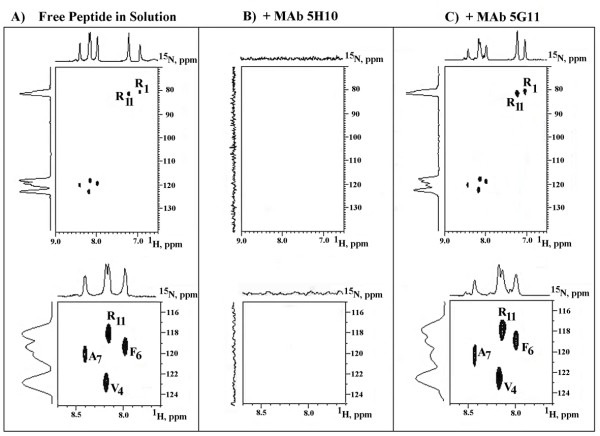
**^1^H-^15^N NMR HSQC spectra and relative 1-D contour plots of the Dsg3_49–60_REWVKFAKPCRE peptide ^15^N-labelled at residues 7, 10, 12 and 14 (A), plus MAb 5H10 (B), or 511G (C)**. Upper panels report portions of the HSQC spectra showing resonances from residues 1, 4, 6, 7, and 11. Lower panels report expanded region of the HSQC spectra showing resonances from the same residues 4, 6, 7, and 11.

The ^15^N-amino acid position in the peptide is reported. Theoretical chemical shift values were derived from  and corrected [29]. Experimental chemical shift values were derived from resonance spectra reported in Fig. 4. The average value of chemical shifts relative to the H and N atoms of α amino residue, and the H and N atoms of η amino residues are reported for Arg-11 (data from: ). The chemical shifts relative to the H and N atoms of α amino residue in Arg-1 are undetectable because of terminal position of the amino acid in the peptide. n.d., not detected.

The resonance spectra of the reaction between MAb 5H10 and the ^15^N-labelled Dsg3_49–60 _REWVKFAKPCRE peptide are illustrated in Fig. 4. Each "spot" in the figure is an NMR signal representing the 1H-^15^N one-bond coupling of the labelled amino acid residues in the peptide. In Fig. 4A, the reported spots correspond to the Arg, Val, Phe and Ala selective ^1^H-^15^N correlation signals of the free peptide in aqueous solution. As already illustrated in Table 1, the expected signals are present and in basic agreement with theoretical data (see BioMagResBank database, ) following sequence-dependent correction of random coil NMR chemical shifts. [29]. The upper panel of Fig. 4A displays the two Arg cross-peak signal, i. e. the chemical shifts relative to the H and N atoms of amino  residues. The lower panel in fig. 4A reports the cross-signal of ^15^N-Arg-11 whereas the ^15^N-Arg-1 was undetectable because of the terminal position in the peptide. Fig. 4B documents that the addition of MAb 5H10 caused the loss of ^15^N-labelled residue spectral resonance signals thus indicating a complete loss of Dsg3_49–60_REWVKFAKPCRE peptide mobility. In contrast to the total signal deletion provoked by MAb 5H10, the chemical shift values of the Dsg3_49–60 _REWVKFAKPCRE peptide remained unaltered following the addition of the anti-Dsg3 MAb 5G11 (Fig. 4C, upper and lower panels).

## Discussion

The present data appear of interest since, as a preliminary consideration, it has to be noted that historically the search for biologically relevant epitopic sequences has demanded and still demands complicated procedures and expensive technologies [30]. Here, the low-similarity principle we used has allowed the utilization of proteomic computational program for the exact epitope definition of a MAb directed towards a 212 amino acid sequence by using only 3 synthetic peptide reagents. Minimally, the screening of a library of at least 14 non-overlapping peptides, each 15 residues in length, would be necessary in the unassisted case. Moreover, the individuation of the Dsg3_49–60_REWVKFAKPCRE motif as the antigenic epitope of the Dsg3 EC1/EC2 domain, aa1-212, might contribute to the understanding of the autoimmune mechanisms in PV. This immunogenic sequence is hosted in the NH_2 _terminal region of Dsg3, which has adhesive function in cadherins [31] and contains the major epitopes recognized by sera from PV patients [32]. Further, chimeric toxins containing the Dsg3 EC1/EC2 domain, aa1-212, have been demonstrated to downregulate anti-Dsg3 IgG-producing B cells in mice immunized with Dsg3 aa1-212 [26].

In a wider context, the epitope mapping approach here described might be helpful in defining peptide antigenicity in a wide spectrum of human diseases including cancer pathologies as well as a range of diverse autoimmune disorders such as insulin-dependent diabetes mellitus, multiple sclerosis, rheumatoid arthritis, and *Pemphigus vulgaris*. The fine profiling of the disease-associated epitopic peptide repertoire is of particular importance in the definition of qualities as antigenicity and immunodominance, and is an essential preliminary step towards effective immunotherapeutical treatments in cancer and autoimmune pathologies. In synthesis, given the caveat that only linear sequences can be defined by the analysis of amino acid motif sharing by two or more proteins, the epitope prediction model we report could form the basis of a rapid, inexpensive and computationally driven system for the individuation of antigenic sequences that are the targets of autoimmune responses. Consequently, this proteomic strategy may serve as a general method suitable to define/distinguish/screen disease-(ir)relevant epitopes within potential autoantigens with clinical application to wide ranging human diseases where the precise targets of self-directed attack are unknown [33].

## Abbreviations

Dsg3, desmoglein 3; EC, extracellular; PVA, *Pemphigus vulgaris *autoantigen; PIR, Protein Information Resource; HSQC, heteronuclear single quantum correlation.
